# Towards an Agreed Labelling System and Protocol for the Diagnosis of Speech Sound Disorder Subtypes in the United Kingdom

**DOI:** 10.1111/1460-6984.70052

**Published:** 2025-05-10

**Authors:** Joanne Cleland, Sam Burr, Sam Harding, Helen Stringer, Yvonne Wren

**Affiliations:** ^1^ Psychological Sciences and Health, Graham Hills Building University of Strathclyde Glasgow Scotland; ^2^ Bristol Speech and Language Therapy Research Unit North Bristol NHS Trust Bristol UK; ^3^ North Bristol NHS Trust Bristol UK; ^4^ Research and Innovation North Bristol NHS Trust Bristol UK; ^5^ King George VI Building Newcastle University Newcastle upon Tyne UK; ^6^ Cardiff School of Sport and Health Sciences Cardiff Metropolitan University Cardiff UK; ^7^ Bristol Dental School, University of Bristol Bristol UK

**Keywords:** assessment, diagnosis, participatory design, speech sound disorder

## Abstract

**Background:**

There is no single classification system or diagnostic protocol for speech sound disorder (SSD). This makes it difficult to collect large‐scale outcome data and determine which interventions work best for which subtypes of SSD. The United Kingdom is unique in that its publicly funded healthcare system allows the collection of such outcome data across large numbers of children; however, a necessary first step towards this is to agree on a consistent diagnostic protocol and classification system for SSD that is feasible for use in the UK healthcare system.

**Aims:**

This study aimed to achieve an initial clinician‐led UK consensus on a diagnostic protocol and classification system for SSD of unknown origin.

**Methods and Procedures:**

A mixed methods participatory design was used. Five UK health services provided SSD paperwork such as local guidelines and protocols for content analysis. Two participatory workshops were used to agree on: (1) a classification system, (2) subtype labels and definitions, and (3) a feasible diagnostic protocol for SSD. The finalised consensus was presented to a national meeting of 283 SLTs to determine the feasibility of the protocol for clinicians across the whole of the United Kingdom.

**Outcomes and Results:**

Workshop participants agreed that the Differential Diagnostic Classification System was preferred for the United Kingdom. A minimum diagnostic protocol, with additional assessment for complex SSD, was agreed. Over 90% of the national SLT meeting agreed that they could implement the definitions and protocol.

**Conclusions and Implications:**

A preliminary diagnostic protocol, classification system, and subtype names and definitions were agreed upon and are broadly in line with those proposed by Dodd (2014). Future work will trial the consensus protocol and classification system in the United Kingdom to investigate treatment outcomes and refine the protocol.

**WHAT THIS PAPER ADDS:**

*What is already known on this subject*

- There are three main classification systems for speech sound disorder (SSD) that are popular globally: the Speech Disorder Classification System (SDCS) (Shriberg et al. 2019); the Differential Diagnostic Classification System (Dodd 2014); and the Psycholinguistic Framework (Stackhouse and Wells 1993). It is not clear which of these, if any, is most used in the United Kingdom. Moreover, previous research suggests that clinicians employ a wide range of different terms for subtypes of SSD and different diagnostic methods to arrive at these subtypes. This lack of consistency, even within the United Kingdom, is confusing for parents, carers, and practitioners and makes it difficult to compare outcomes.

*What this study adds*

- We showed that most clinicians in the United Kingdom use the Differential Diagnostic Classification System (Dodd 2014). We therefore suggest that this is now used consistently in the United Kingdom, with some modifications. A feasible diagnostic protocol which includes using the assessment designed specifically for this classification system, the Diagnostic Evaluation of Articulation and Phonology (Dodd et al. 2002), was agreed for clinical use.

*What are the clinical implications of this work?*

- Clinicians in the United Kingdom can use the subtype labels and diagnostic protocol described here to diagnose subtypes of SSD in a consistent manner. Children with more complex SSD or concomitant disorders will require additional assessments.

## 1 Introduction

Speech sound disorder (SSD) is an umbrella term for any difficulty acquiring the sounds of the ambient language. This encompasses one or more difficulties with perception, articulation (motor production), phonological representations and/or prosodic difficulties (McLeod and Baker 2017). SSD is a high‐prevalence condition, estimated at 3.4% to 3.8% of children aged 4–8 (Eadie et al. 2015; Shriberg et al. 2019; Wren et al. 2016), and with upwards of 76,000 children referred for services in the United Kingdom annually (Broomfield and Dodd 2004). When SSD persists into the school years, it is associated with reduced educational, social, emotional, and behavioural outcomes (Wren et al. 2023; Wren et al. 2021).

SSD is a theory‐neutral term, likely comprising subtypes that arise from different aetiologies and with different behavioural manifestations requiring different treatment approaches. While the use of the umbrella term is convenient, it can also be problematic in that it may be difficult to predict which children are most likely to have persistent SSD and therefore which children should be prioritised for treatment (Wren et al. 2016). Treatment begins with two main clinical reasoning processes on the part of the speech and language therapist (SLT) (Diepeveen et al. 2020). First, a diagnostic process to determine whether a child presents with an SSD and, if so, which subtype; and second, therapeutic reasoning to decide which intervention, if any, to choose. These processes are linked. Most interventions are designed by their originators to treat specific subtypes of SSD (Wren et al. 2018). For example, (conventional) minimal pair intervention was originally designed for children who display a loss of phonemic contrast in their speech, usually known as a phonological SSD subtype (McLeod and Baker 2017), whereas children with motor production difficulties will need motor‐based approaches (McLeod and Baker 2017). However, there is a lack of consensus in the literature on labelling and indeed on clear descriptors for any labels. While some SSDs are attributable to medical or genetic conditions, for example, cleft lip ± palate, cerebral palsy, or Down syndrome, the majority, to date, remain of unknown origin (Shriberg et al. 2010). Children with SSD of unknown origin are the most commonly referred to SLT services (Broomfield and Dodd 2004) and are considered here.

In their textbook on SSD, McLeod and Baker (2017) list 41 different terms used in the literature to describe SSDs of unknown origin. Studies asking clinicians which labels they typically use do not also ask them to give information about how they operationalise these labels or which labels they consider synonymous. For example, a recent mixed‐methods study (Diepeveen et al. 2020) recorded 35 different terms used by SLTs in the Netherlands to describe SSDs. Although the SLTs gave broad characteristics for categories of SSD, for example, ‘phonological disorder/delay’ (33 different labels) was characterised by features such as ‘simplification processes, frustration, often unintelligible speech’, the SLTs did not give precise definitions for individual terms, and there was much overlap between diagnostic categories. This makes it difficult to determine whether or not terms like ‘phonological impairment’ are synonymous with ‘phonological disorder’ or ‘phonological delay’. Lack of consensus is problematic for clinicians because it makes it difficult to determine which interventions are most appropriate or what the prognosis for discrete subtypes of SSD might be. It also makes it difficult for researchers to conduct studies with homogeneous groups and to measure outcomes. Importantly, it is also confusing for parents who wish to seek advice and guidance relevant to their own child's specific diagnosis. There is therefore a need to agree on labels that have consistent definitions that clinicians understand and can implement in their own clinical practice.

Although there are many different terms used to describe SSDs, there are fewer classification systems. Waring and Knight (2013) provided a review of three commonly used SSD classification systems (Waring and Knight 2013): the Speech Disorder Classification System (SDCS) (Shriberg et al. [2010] and updated in Shriberg et al. [2019]); the Differential Diagnostic Classification System (Dodd 2014) and the Psycholinguistic Framework (Stackhouse and Wells 1993). The Psycholinguistic Framework views children's speech difficulties as breakdowns at the levels of input, representation, and/or output. It is therefore useful for intervention planning for individual children, but less so for subcategorising cases of SSD for research purposes, nor does it provide useful diagnostic labels for parents and carers.

The Differential Diagnostic Classification System (often referred to as Dodd's system) is also based on a psycholinguistic model of speech production; however, it provides specific subtype labels: phonological delay; consistent atypical phonological disorder; inconsistent phonological disorder (IPD); articulation disorder; and childhood apraxia of speech (CAS—sometimes known as developmental verbal dyspraxia or DVD). These labels are designed to be clinically useful to SLTs as most map to specific interventions. In contrast, the SDCS is an aetiology‐based system designed for research purposes. The most recent version of this system includes the terms: speech delay‐genetic; speech delay‐otitis media with effusion; speech delay‐developmental psychosocial involvement; speech errors‐/s/; speech errors‐/r/; motor speech disorders‐dysarthria; motor‐speech disorders‐CAS; and ‘speech motor delay’ (Shriberg et al. 2019). Notably absent from Dodd's system is the motor speech disorder label ‘dysarthria’. This is because her system focuses on SSD of unknown origin, and in most cases of dysarthria a cause is known, for example, cerebral palsy. Of these three systems, the Differential Diagnostic Classification System and the SDCS are most widely used internationally (Terband et al. 2019).

It is likely that the choice of which classification system to use differs geographically and is influenced by training programmes in each country. Within the United Kingdom, the professional body, the Royal College of Speech and Language Therapists, sets curriculum guidelines for higher education institutions (RCSLT 2021). Although these guidelines specify that subtyping of SSD should be on the curriculum, no particular classification system is mandated. However, anecdotal evidence suggests that many SLTs in the United Kingdom use Dodd's (2014) classification system, and the terms used within the curriculum guidelines are more closely aligned with these than those in the SDCS (Shriberg et al. 2010).

### 1.1 SSD Assessment Procedures

A thorough SSD assessment is multifaceted. Macrae (2016) suggests that assessment should include standardised single‐word testing, additional single‐word testing designed to look at each child's difficulties in‐depth, a connected speech sample, stimulability testing, and an assessment of inconsistency. Similarly, the Child Speech Disorder Research Network (the United Kingdom and Ireland) suggests that speech samples include single‐word testing of at least 100 words, connected speech sampling, and, again, an additional wordlist designed to look at a child's difficulties in detail. Surveys of SLTs suggest that some, but not all, of these are included by clinicians in their diagnostic toolbox. For example, in a survey of 333 SLTs in the United States, Skahan et al. (2007) reported that over 85% of clinicians used standardised single‐word tests, stimulability testing, and an estimate of intelligibility, ‘always or sometimes,’ when assessing children with SSD. In Australia, 231 SLTs surveyed by McLeod and Baker (2014) also reported that one of 14 different single‐word tests was used by at least one SLT in the sample, in addition to most SLTs (58% always and 26% sometimes) using connected speech samples, stimulability testing (78% always), and an estimate of intelligibility (55% always).

### 1.2 SSD Differential Diagnosis

The assessment procedures above should give SLTs enough information to make a differential diagnosis. However, this process is not straightforward, in part because SSD subtypes may overlap, and while studies have asked what subtype labels clinicians use, they have not asked how they operationalise definitions in practice. This is important because diagnostic definitions can, and do, change over time (Maitland 2010), and there is a tension between how diagnoses are described in the literature and how clinicians apply them. Dodd has been influential in trying to solve this problem by designing a standardised assessment, the DEAP (Dodd et al. 2002), which is designed to map to her subtypes. However, as Waring and Knight (2013) point out, ‘*presently evidence is cited about test–retest reliability and inter‐rater reliability; however, how well all clinicians using the DEAP arrive at the same diagnosis is unknown*’ (33). Moreover, many studies purport to use this or another classification system but do not use the specific assessments designed for this purpose. For example, it is not clear in the original study by Broomfield and Dodd (2005) how children were assigned to diagnostic subtypes, and the pre‐ and post‐intervention assessment measured only consistency on a list of 25 words. Moreover, no children in this study received a diagnosis of CAS. This under‐representation of motor involvement is further highlighted by a data‐driven study of 150 Dutch children with SSD which used a wide test battery to show that children tend to cluster into three groupings: phonological deficit, phonological deficit with motoric deficit, and severe phonological and motoric deficit (Diepeveen et al. 2022).

Moreover, a recent study looking at testing the validity of Dodd's model used a different assessment battery, for example, including the Goldman‐Fristoe (Goldman and Fristoe 2000) as the test of single‐word naming, to categorise children into Dodd's subtypes (Ttofari Eecen et al. 2019). While they found similar prevalence rates to other studies for each specific SSD subtype, they differ from, for example, Broomfield and Dodd (2004) in that they did not find any children with isolated articulation disorder but did find 10% of the sample had ‘suspected atypical speech motor control’.

Clearly, then, while assessments such as the DEAP are proprietary, the diagnostic labels are not. Moreover, how well definitions of diagnostic labels work in practice in real‐world settings is not known. Clinicians may apply these labels after using any assessment protocol they choose, and it is at present unknown how well clinicians agree on differential diagnoses in real healthcare contexts. An approach which involves clinicians in operationalising differential diagnosis and deciding which assessments to prioritise for this process is key.

Diepeveen et al. (2020) suggest that SLTs’ main motives for deciding what to include during diagnostic testing are time and ease of use. Although an international consensus on classification and a diagnostic protocol is desirable (Waring and Knight 2013), this study focuses on establishing a classification system and diagnostic protocol acceptable and feasible for SLTs working within the publicly funded, free at point of access, National Health Service (NHS) in the United Kingdom. Our aim is to create standardised SSD labels and a diagnostic protocol that reflects current practice and is feasible for clinicians working in this context. By reflecting current practice, we can better ensure implementation. We define feasibility in our study using the APEASE criteria (Acceptability, Practicability, Effectiveness, Affordability, Side‐effects, and Equity [Michie et al. 2014]), which incorporates both practical considerations and safety and effectiveness. We limit our study to the United Kingdom for several reasons. First, time available to undertake assessment and ease of use/familiarity will differ between healthcare systems. Secondly, specific assessments are standardised on specific populations and with speakers of specific languages; and lastly, the UK NHS service is unique in that there is potential to collect large‐scale data from every patient in the United Kingdom that accesses the service, enabling us to, in the future, answer questions about treatment effectiveness in children with SSDs.

To ensure a classification system and diagnostic protocol are fit for purpose within this context, they must be co‐designed with the clinicians and services who are going to use them. We therefore employed a participatory design, focused on involving end users of the SSD subtype labels and diagnostic protocols (Roper and Skeat 2022), which in this case are SLTs. In addition, we sought to ensure that any labels used should be sensitive to the needs of parents and carers. Moreover, it is important that the tools used in any diagnostic protocol are acceptable to both parents and children. We therefore chose a participatory design with practising clinicians, rather than any expert panel approach such as a Delphi. Indeed, it should be noted that there is already a consensus in English‐speaking countries across the world that the umbrella term for difficulties with speech production in children is ‘speech sound disorder’ of unknown or known origin (see, for example, USA: https://www.asha.org/practice‐portal/clinical‐topics/articulation‐and‐phonology/ and UK: https://www.rcslt.org/related/categories/topic‐area/speech‐sound‐disorders/). This differs considerably from the controversy that formerly surrounded childhood language impairments, where an international consensus exercise using Delphi methodology was necessary to arrive at the new term – Developmental Language Disorder (Bishop et al. 2016). Second, this work comprises part of a larger project where we aim to collect large‐scale outcome data for children with SSD in the United Kingdom. To do this, we need to first standardise existing diagnostic practice in order to make any diagnostic processes immediately implementable. Future work will trial the consensus labels and protocol, and changes are anticipated.

### 1.3 Aims

The overarching aim of this study was to achieve an initial consensus on which classification system for differential diagnosis is most appropriate for use in the United Kingdom. To achieve this, we had the following objectives:

1. To agree on an SSD classification system which reflects current clinical practice in the United Kingdom, supports the clinical decision‐making process, and is appropriate for large scale adoption.
2. To agree on definitions for each subtype of SSD which are clinically relevant for the UK context.
3. To agree on a diagnostic protocol for categorising children with SSD into these subtypes, which is feasible for the publicly funded NHS in the United Kingdom.

## 2 Method

### 2.1 Participatory Design

Participatory designs democratise the development process by involving the end users of the product or system (Roper and Skeat 2022). This contrasts with the traditional approach of experts, usually researchers, designing a classification system based on their own theoretical position or researchers suggesting diagnostic protocols without considering feasibility within a clinical context. This more traditional approach can lead to problems implementing research because clinicians have different challenges and priorities from researchers (Douglas et al. 2023). Participatory designs can lead to quicker implementation in clinical practice. This study used mixed methods. First, a content analysis of local paperwork/guidelines on how SSDs are diagnosed and classified in different SLT teams within the United Kingdom was undertaken. Second, two participatory workshops with SLTs representing these teams and a parent of a child with an SSD, and lastly stakeholder engagement via short anonymous polls with SLTs attending the national Clinical Excellence Network (CEN) for Speech Sound Disorders annual meeting (June 2023). These stages of the study took place sequentially. That is, the content analysis was completed prior to the workshops and was presented at the workshops for discussion. Following both workshops, the overall decisions made by workshop participants were presented at the CEN meeting to gauge agreement, but no changes were made at this last stage.

#### 2.1.1 Ethics

Ethical approval for this study was obtained from the study sponsor, Bristol NHS Trust. Full approval for the study was obtained from the NHS Health Research Authority (22/HRA/1962) prior to recruitment.

#### 2.1.2 Participants

SLT teams were recruited via invitation to specific NHS providers (i.e., services) that had expressed an interest in participating in a larger project designed to establish the most effective care pathways for children with SSD. Five NHS services in England (*n* = 4) and Scotland (*n* = 1) took part in providing local service‐relevant paperwork, and six individual SLTs from these five NHS services consented to participate in the workshops. Services were selected to be representative of the 42 integrated care boards in England and the 14 health boards in Scotland in that they represented a variety of urban, semi‐urban and accessible‐rural locations providing for patients of all socioeconomic statuses and served populations between 460K and around one million (see table one). The services employed between 11 and 115 full‐time or part‐time SLTs. SLTs were selected to represent their service by their managers because they had specific responsibility or expertise in SSD. One service put forward two SLTs to take part in the workshops as both SLTs had a particular remit for SSD but worked in different teams. All of the SLTs were practitioners who diagnosed and treated SSD regularly. The practitioners were all female, aged 34–60, had between nine and 39 years of experience as an SLT, and their caseloads were 26% to 90% SSD. Two parents (both mothers) of children with severe SSD and members of the project patient public involvement group were invited to join. One was able to attend and participate in the workshop as an expert by experience to support the study. The CEN meeting was held after the participatory workshop results had been analysed. It was open to any SLT in the United Kingdom who was interested in SSD and was attended by 283 clinicians via Zoom. Separate work reports the views of children and parents attending community SSD clinics (Harding et al., unpublished manuscript).

#### 2.1.3 Content Analysis of Local Paperwork and Guidelines

Prior to the workshops, participants were asked to submit via email all diagnostic criteria, care pathway information or clinical decision‐making resources used by SLTs working with children with SSD in their service. These documents were subjected to directive content analysis (Hsieh and Shannon 2005). That is, we used our a priori knowledge from the literature above to identify:

1. Which, if any, classification systems were teams using?
  - Taking as a starting point the three classification systems described in Waring and Knight (2013), documents were searched for mentions of these or other systems (e.g. ‘Dodd's system/ Differential Diagnostic Classification System’, ‘Shriberg/ SDCS’, psycholinguistic, other mentions of ‘classification’) and counted and tabulated.
2. How, if at all, were the subtypes of SSD described and defined?
  - Taking as a starting point the SSD labels described in McLeod and Baker (2017), documents were searched for mentions of SSD subtypes or labels (e.g., phonological delay/disorder/impairment, articulation delay/disorder/impairment etc.) with definitions and were counted and tabulated.
3. Which assessments (published or locally designed) were the teams using and how, if at all, did they lead to diagnosis of specific subtypes of SSD?
  - Documents were searched for mentions of any named assessment and counted and tabulated. For example, any use of the word ‘assessment’ followed by a reference or link.

Content was tabulated and similarities and differences were identified. These results were then presented for discussion at the participatory workshops.

#### 2.1.4 Participatory Workshops

Two 3‐h workshops were held online using Microsoft Teams. The workshops were held 1 week apart to allow participants to reflect on the discussions. All participants attended both workshops. The workshops were chaired by two members of the study team (Cleland and Burr), who are academic SLTs who specialise in SSD. Their role was to chair the meetings and take notes, not to contribute to the discussion or decision‐making. Two other members of the study team (Harding and Wren) observed the meetings but had their cameras and microphones off throughout. Field notes were taken during the workshops, and they were recorded and transcribed using the speech‐to‐text function in MS Teams. The second author quality‐checked the automatic transcription and corrected errors by reviewing the meeting recordings.

#### 2.1.5 Workshop 1 Structure

The workshop agenda was presented via PowerPoint presentation as a set of tasks: (1) to agree on SSD subtypes; (2) to agree on an SSD diagnostic protocol. To achieve this, the results of the content analysis were first shown to participants. They were encouraged to give opinions about how any potential subtype definitions would work for their service. Each subtype was taken in turn, and the definitions (if any) from the content analysis were presented and discussed. Once subtypes were agreed, the content of diagnostic pathways was discussed, with a focus first on screening and then on differential diagnosis. We considered which aspects of speech should be assessed (single words, connected speech, etc.); which stimulus tools/assessments should be used; and whether the suggestions were feasible within the NHS context and acceptable to families and SLTs. Participants were encouraged to contribute either verbally or by writing their ideas on an online interactive whiteboard (Jamboard).

#### 2.1.6 Workshop 2 Structure

Again, the workshop agenda was displayed via PowerPoint as a set of tasks: (1) to recap on what was agreed in the first workshop; (2) to decide the final aspects of the diagnostic protocol. The agreed subtype definitions and then the diagnostic protocol were presented via PowerPoint to participants for comment and refinement. Again, participants could contribute either verbally or by writing their ideas on an online interactive whiteboard (Jamboard).

#### 2.1.7 Post Workshop Data Checking

A report of the workshops was sent to participants 6 weeks after the final workshop for checking and confirmation of accuracy.

#### 2.1.8 Stakeholder Engagement

**FIGURE 1:**
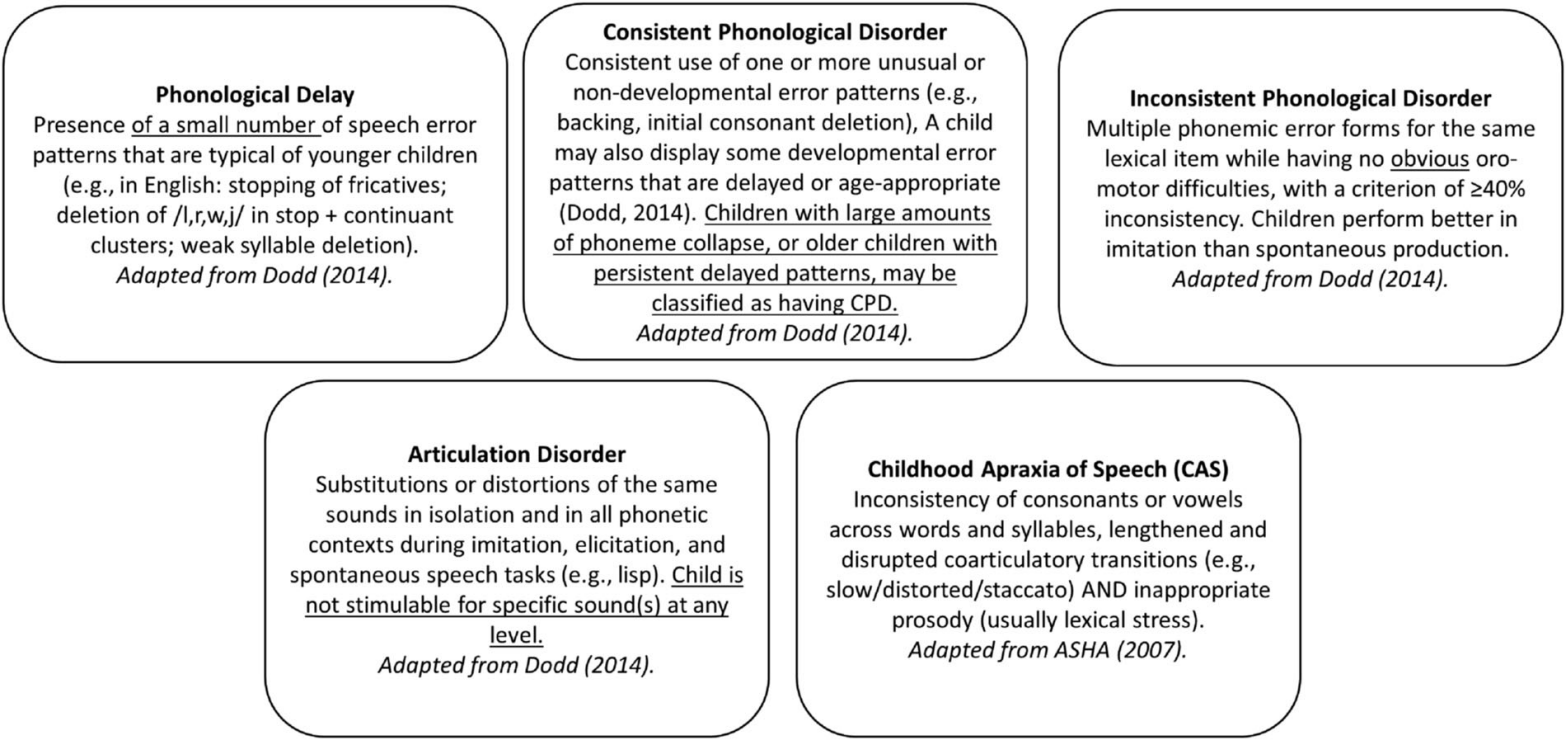
Agreed SSD subtype definitions with changes to the wording in Dodd (2014) underlined. *Note*: The CAS definition comprises the three core consensus criteria for the condition.

**FIGURE 2:**
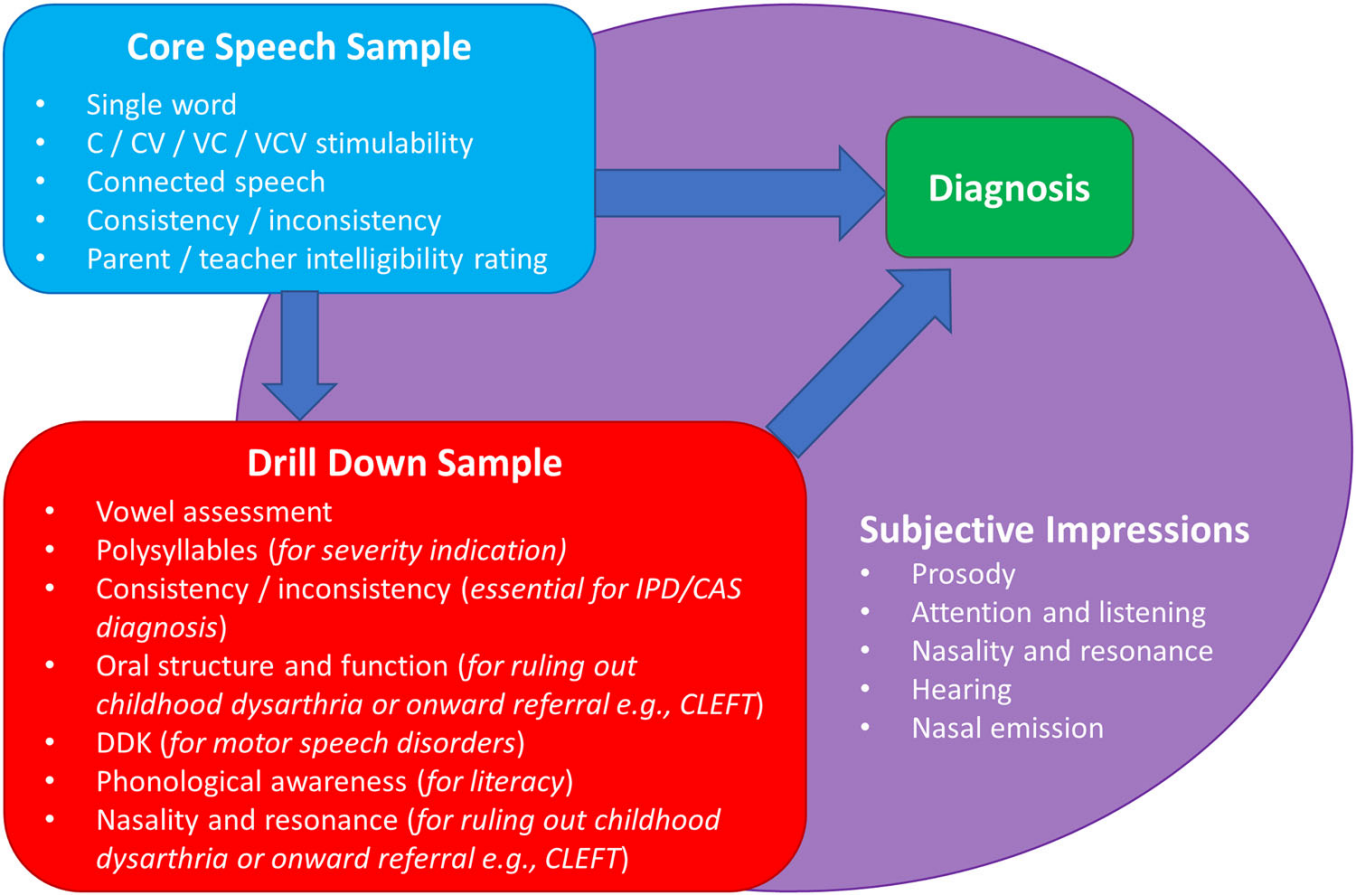
Diagram showing the minimum diagnostic protocol. The ‘Core Speech Sample’ (blue) should be completed for every child with a suspected SSD. The ‘Drill down sample’ (red) is for children with more complex SSD, or where onward referral might be necessary (shown in brackets). The ‘subjective impressions’ (purple) details the other areas the SLT should assess from interaction and conversation with the child.

Attendees at the National Clinical Excellent Network for SSD engaged with presentations explaining the results of the above workshops, including the agreed subtypes, their definitions, and a diagnostic protocol. All authors of this paper gave presentations regarding the wider research project, and the specific presentation on classification and subtypes was given by the first author. All figures in this paper were presented to attendees. Throughout the presentations attendees were invited to ask questions using the Zoom chat function or verbally. The were invited to respond anonymously to three polls asking:

1. Which classification system do you (normally) use:
  - Dodd's Differential Diagnostic Classification System
  - Shriberg's SDCS
  - Stackhouse and Wells’ Psycholinguistic Framework
  - International Classification of Functioning, Disability and Health (ICF) (NB this classification system can be used in combination with other systems, we include it here for completeness).
  - Other/ do not use a system
2. Do you agree with the Definitions? (Figure 1 was presented and explained)
  - Yes
  - No
3. Do you think you could implement the definitions and protocol? (Figures 1 and 2 were presented and explained, and the protocol below was explained)
  - Yes, easily
  - Yes, with difficulty
  - No

## 3 Results

Results are organised first with tabulated details of the content analysis and then in relation to each of our above aims. This is followed by a section on data checking and then finally stakeholder engagement to reflect the chronology of the study.

### 3.1 Content Analysis of Local Paperwork and Guidelines

Data received from the five NHS sites varied from brief summary guideline documents to service‐level policies for SSD populations and documents to support the clinical decision‐making process for SSD diagnosis and intervention selection (Table 1). The number of documents received from each SLT service varied across the five sites (range 1–11).

**TABLE 1:** Service demographics content analysis results.

NHS Site	Population	English speaking %	No. files	SSD guidance	References	Pathway flowchart	Decision making tool	SSD subtype definition	SSD subtype diagnostic criteria	Assessment tool information	Assessment selection
**A**	916 310	99	4	✓	✓	✓	✓		✓		✓
**B**	906 300	97	11		✓	✓	✓	✓	✓	✓	
**C**	529 000	98	1			✓	✓				
**D**	762 800	92	6				✓				
**E**	457 100	88	2							✓	

*Note*: % English Speaking = “speaks English well or very well” (Scottish census question) or “English as a main language” (England census question).

Differences were observed in the content of the documentation provided by the NHS services (Table 1). Inclusion of a clinical decision‐making tool to support SSD diagnosis and/or intervention selection was the most common element in the data, evidenced by four of the five participating NHS services. Clinical guidance on assessment, diagnosis, and intervention for children with SSD, definitions of SSD subtypes and information on appropriate assessment selection were, in each case, provided by only one of the five participating services. Table 2 summarises the content of these documents in relation to each aim of the study.

***Aim 1: To agree on an SSD classification system that reflects current clinical practice in the United Kingdom, supports the clinical decision‐making process, and is appropriate for large scale adoption***.

**TABLE 2:** Content analysis results.

NHS site	Classification systems	Subtype definitions	Assessments
**A**	DDCS (Dodd, various dates)	Dodd's	No standardised assessments: single word naming, connected speech, intelligibility, and oromotor assessment
**B**	DDCS (Dodd, various dates)	Dodd's	DEAP, STAP, NDP, ICS, GOS.SP.ASS
**C**	DDCS (Dodd, various dates)	Not specified	Not specified
**D**	DDCS (Dodd, various dates)	Dodd's	Locally designed single word naming test and narrative connected speech tool
**E**	DDCS (Dodd, various dates)	Dodd's	DEAP, STAP, CLEAR, NDP

Abbreviations: DDCS = Differential Diagnostic Classification System (Dodd 2005, 2014); DEAP = Diagnostic Evaluation of Articulation and Phonology (Dodd et al. 2002); STAP = South Tyneside Assessment of Phonology (Armstrong and Ainley 1993); NDP = Nuffield Dyspraxia Programme (Williams and Stephens 2004); ICS = Intelligibility in Context Scale (McLeod et al. 2012); GOS.SP.ASS = Great Ormond Street Speech Assessment (Sell et al. 1999); CLEAR = Clear Phonology Screening Assessment (Kerryjane and Spilsby 2006).

The content analysis and Workshop 1 revealed that all services were using the SSD classification proposed by Dodd (2014). It was agreed that this classification system is appropriate for use in the United Kingdom and likely to be familiar to most SLTs practising in the United Kingdom. However, the content analysis revealed that although the overarching system was in use across all sites, there was variation in whether subtype definitions were given and some inconsistency in the labels used (for example, the use of ‘articulation delay,’ which is not a term used by Dodd). Aim 2 therefore considered reaching consensus on subtype labels and definitions.

***Aim 2: To agree on definitions for each subtype of SSD which are clinically relevant for the United Kingdom***.

Following the consensus to use the Differential Diagnostic Classification System (Dodd 2014), some amendments to the definitions of the sub‐types were proposed by participants and agreed upon. The definitions are shown in Figure 1, with amendments underlined. These amendments were necessary to fit with how the clinicians operationalise these definitions in clinical practice. For those children who may not fit neatly into one subtype, the group agreed to prioritise an initial diagnosis that would allow treatment planning with consideration that a child's presentation may change over time. For example, children who present with phonological patterns alongside some articulation difficulties might be initially classified as having a phonological disorder, and over time, this may resolve to residual speech sound errors which could be reclassified as an articulation disorder. Participants agreed that giving children a mixed SSD diagnosis could be overly complicated as well as confusing for parents. This was agreed to by the parent representative.

### 3.2 Articulation Disorder

There was discussion surrounding the idea that an articulation disorder should involve a motoric or phonetic difficulty. Although the original definition suggests that any difficulty producing specific sounds is included within ‘imitation and elicitation’, the group agreed that clarification should be added that this means children are not stimulable for these specific sounds at any level (e.g., C, CV/VC).

### 3.3 Phonological Delay

Participants discussed the severity and persistence of a phonological delay and at what point, if any, a delay should be considered a disorder and indeed whether ‘phonological delay’ and ‘phonological disorder’ are distinct subtypes. The group decided to keep delay and disorder as distinct subtypes, with the caveat that a minor amendment was added to specify that the number of delayed speech error patterns observed should be small. The group discussed whether it would be possible or appropriate to specify a number of typical error patterns which constitutes ‘small’. While the group did agree that it was likely to be only two to three, the decision was made not to specify a number because of the need to consider the impact of multiple factors, such as the combination of patterns, the child's age, and the overall impact on intelligibility. The group agreed that children with system‐wide contrast collapse were likely better served by a phonological disorder diagnosis.

### 3.4 Consistent Phonological Disorder (CPD)

The amended definition specifies, in addition to unusual or non‐developmental phonological process, that children with significant phoneme collapse or older, school‐aged children with persistent delayed error patterns may also be categorised as having CPD. It is worth noting that this is contrary to Dodd's original definition, where older, school‐aged children with only delayed patterns would be diagnosed with ‘phonological delay’. This will be explored further in the discussion.

### 3.5 IPD

There was discussion around whether children with this diagnosis could present with mild or subclinical oro‐motor difficulties, and it was agreed to add ‘no obvious’ to the definition to add clarity. It was agreed that, for clarity, the 40% inconsistency criterion should be amended to 40% or more using the ‘≥’ symbol.

### 3.6 Childhood Apraxia of Speech (CAS)

Dodd's original definitions used the term ‘developmental verbal dyspraxia’. The study team introduced, in line with the international consensus, the term ‘childhood apraxia of speech’, and discussion was had around the adoption of this term. Participants agreed with this terminology change. Given the change in terminology and acknowledging the increase in the research literature, and particularly new treatment approaches on this disorder over the last decade, participants agreed to use the American Speech‐Hearing Association (ASHA) three core criteria for the definition of CAS instead of Dodd's definition.

***Aim 3: To agree on a diagnostic protocol for categorising children with SSD into these subtypes which is feasible for the publicly funded NHS in the United Kingdom***.

Only two services provided assessment/protocol information for how to differentially diagnose children with SSD (content analysis). Given the range of assessment materials suggested in Table 2, discussion focused first on what information was required for a diagnosis (e.g., types of speech samples) before moving onto which tools should be used to collect this information. Participants agreed that the first step in diagnosis is confirming the presence/absence of an SSD and that some parts of screening may happen prior to direct contact with an SLT, that is, via a telephone helpline or referral from, for example, a teacher. The development of a screening protocol for SSD was discussed and debated by workshop participants. It was agreed that a measure of intelligibility (e.g., using the Intelligibility in Context Scale, ICS [McLeod et al. 2012]) is useful to evaluate the impact and severity of a child's speech difficulties on their communication with the people around them. Participants agreed that, while some therapists may complete the ICS with parents or families as part of assessment, many services use the ICS as part of the referral process and documentation from education settings. In both cases, it was agreed that the ICS provides useful screening information about a child's speech that informs prioritisation of care and assessment. Participants also agreed that the screening protocol should include informal conversation with the child to provide a subjective impression of speech. Where children are reluctant to engage in conversation, a structured connected speech screen can provide an impression of speech skills. Participants’ clinical perspectives aligned with the growing body of evidence that collecting connected speech data is important to better understand where the level of breakdown occurs for different children with SSD:

“at the simplest level, also I think assessing connected speech is so important for those children who are referred who are unintelligible. But when you assess it (at) single word level they're fine. And actually, it's almost like, ‘is there a breakdown at this level? Yes / No. Is that because of XYZ?’, rather than actually transcribing the data.” (SLT1, Workshop 1)

Participants discussed the types of speech sample data that would need to be collected to make a diagnosis for each and any of the subtypes. It was agreed that different levels of data would be required, but that there should be a common ‘core’ speech sample collected for all children. This core sample includes single words, consonant (C), consonant‐vowel (CV) and vowel‐consonant (VC) stimulability, connected speech, and parent/carer/teacher ratings of the child's intelligibility. The inclusion of consistency/inconsistency was discussed, but it was agreed that a full inconsistency assessment would not be required for all children and would instead be screened initially and be part of a more in‐depth or ‘drill down’ sample required for children where there is diagnostic uncertainty (see ‘inconsistency stimulus tools below’). The need to allow for the impact that different levels of clinical experience may have on the speed of the diagnostic process was discussed regarding the protocol, and it was acknowledged that assessment and diagnosis may take more than one clinical session.

*“it's about the speed at which you think, and for a lot of staff, it'll take them a bit of thinking to realise inconsistency as somewhere they should go and therefore having it in a sort of second session or whatever is OK. But some of us might decide really quickly we're going to do that inconsistency assessment, but it doesn't mean that everybody has the experience to do that. So I think it is fine in the drill down” (SLT2, Workshop 2)*

Although the group acknowledged the benefits to research of having larger datasets for each child, the need to minimise burden of assessment for children, families, and clinicians was agreed upon by participants.

### 3.7 Stimulus Tools and Assessments

#### 3.7.1 Single Word Assessments

Two services suggested published speech assessments such as the DEAP (Dodd et al. 2002) or DEAP Toddler Phonology Test (McIntosh and Dodd 2011); the ICS (McLeod et al. 2012); the South Tyneside Assessment of Phonology (Armstrong and Ainley 1993); the CLEAR Phonology Screen (Kerryjane and Spilsby 2006); and the Nuffield Dyspraxia Programme assessment (Williams and Stephens 2004) in their guidelines. Of these, the South Tyneside Assessment of Phonology and the CLEAR are single‐word speech assessments. The Nuffield Dyspraxia programme is primarily a single word assessment but also contains phrases. The DEAP is also mainly a single‐word assessment but incorporates stimulability testing, inconsistency assessment, a limited assessment of connected speech, and a short oro‐motor assessment. No service stipulated that a particular assessment must be used. The participatory workshop discussed the assessments and stimulus tools with regard to clinical experience, evidence base, and UK standardisation, using the APEASE (Acceptability, Practicability, Effectiveness, Affordability, Side‐effects, and Equity) criteria (Michie et al. 2014) to guide discussion. The issue of the cost of assessment materials in particular was discussed at length, and it was agreed that, because of funding differences across services, some flexibility would have to be built in in order to make implementation in clinical services feasible.

In terms of the single‐word assessment tools, participants agreed that the DEAP (Dodd et al. 2002) was a preferred method of assessment. This was because the DEAP is standardised on a UK population and offers subtests designed to differentially diagnose within Dodd's framework (Dodd 2014). However, it was agreed that this could not be universally specified as a required stimulus tool because of the financial implications of making the assessment available to all services and staff. Nevertheless, participants agreed to include the DEAP (Dodd et al. 2002) as a suggested tool for single‐word assessment. The STAP (Armstrong and Ainley 1993) was discounted due to participants feeling it is outdated. Some participants felt that using the CLEAR (Kerryjane and Spilsby 2006) was a false economy because, although the initial outlay for the tool was lower than the DEAP, the cost of clinical time to analyse and interpret the results negated any significant cost saving over time compared with the DEAP, which offers a framework for identifying phonological processes.

*“Let's think about how much time you have to spend analysing and OK face to face with the child, …it might appear quicker, it might be cheaper to buy the assessment. But in terms of how hard you then have to work to do the analysis on the CLEAR, you have to spend a lot more time organising how you're going to pull together that data. And you know when you've got something like the DEAP and, it's phonological processes. It's a very quick run through that. So it appears cheap the CLEAR because it's cheap to buy the assessment.” (SLT1, Workshop 1)*

The single word section of the Nuffield Dyspraxia Programme (Williams and Stephens 2004) was agreed to be a tool preferred as part of the drill‐down criteria if CAS were suspected, and it was agreed it is not designed for children with phonological disorders.

#### 3.7.2 Assessing Stimulability

Participants acknowledged that stimulability would often be assessed informally by SLTs, with some specific tools being used to assess specific sounds that had not been elicited during single‐word or connected speech assessment and which required closer examination. Participants agreed that the Stimulability Assessment (Miccio and Williams 2021; Powell and Miccio 1996) offers a broad range of contexts for examining specific sounds, and as a free resource, it may be more appealing and accessible to NHS services, but that other tools, such as the DEAP articulation assessment stimulability section (Dodd et al. 2002), could also be used. The group agreed stimulability of every consonant should not be assessed; rather SLTs should focus on specific sounds absent from the child's inventory following phonological analysis (e.g., using the DEAP).

#### 3.7.3 Connected Speech Stimulus Tools

While it was agreed that connected speech is important, participants discussed what SLTs would do with the connected speech sample after it was collected and how much analysis was required to make a diagnosis. Challenges with the DEAP connected speech assessment, which comprises three composite pictures for children to describe, were discussed. Anecdotally, the workshop participants found that children often listed the individual items on the composite pictures rather than producing natural, connected speech. Participants agreed that using an expressive language assessment to collect connected speech would be a useful way to simultaneously assess for any concomitant language difficulties. It was agreed that a structured format for eliciting connected speech was preferable to an informal conversation because, for children with lower intelligibility, it provides a context and targets to help the SLTs identify the child's intended target words. On this basis, the Renfrew Action Picture Test [RAPT; (Renfrew, 2016)] is a suggested tool for the protocol. Rather than formally analysing this connected speech sample, the group agreed that an informal, tick‐box type approach to provide a sense of the characteristics of a child's speech skills listed under ‘subjective impressions’ (Figure 2, purple) would be appropriate to provide enough initial information.

#### 3.7.4 Intelligibility Rating Stimulus Tools

Participants agreed that the ICS (McLeod et al. 2012) is appropriate to recommend as a parent/carer reported measure of impact because it is quick to administer, free, and widely available in a variety of languages.

#### 3.7.5 Inconsistency Stimulus Tools

The group discussed the use of the DEAP inconsistency assessment (Dodd et al. 2002) and acknowledged the challenge of asking children to repeat 25 items three times. The use of 10 untreated words was discussed as a quick and simple tool to use to screen for inconsistency (Crosbie et al. 2021). Using this as a tool to generate a definitive diagnosis was seen as challenging because there is no evidence to support this. Consideration was given to the suggestion that administering the DEAP diagnostic screen for consistency and then using the cut‐off of ≥ 50% inconsistency to trigger further full assessment using the DEAP inconsistency subtest. This was agreed on the basis that it would be achievable with all children from a time perspective, compared with the full DEAP inconsistency assessment (Figure 2).

### 3.8 Analysing Speech Data

There was little guidance in the content analysis or the workshops on how SLTs might arrive at a final differential diagnosis from the selected tools. However, given the SLTs selected Dodd's classification system and her assessment, the DEAP, there was the implication that the DEAP process be followed to arrive at a diagnosis. For intervention planning, particularly target selection, SLTs need to be mindful of how they analyse assessment data. Participants discussed the use of the freely available Phonetic and Phonological Systems Analysis (PPSA) (Bates and Watson 2012), which is in use in some services. While some participants felt that therapists conducted this type of analysis without the specific use of the published tool itself, it was agreed that the tool did support accurate phonological analysis, which is necessary for subsequent target selection for interventions. This analysis framework also allows SLTs to identify delayed or disordered phonological patterns, which is a necessary step in differentiating phonological delay and phonological disorder. Participants discussed and agreed that intelligibility ratings obtained from the ICS (McLeod et al. 2012) are important for determining severity and measuring change.

### 3.9 Summary of Recommended Tools

Table 3 details the suggested tools participants agreed could be used to achieve the core sample (Figure 1, blue). These tools additionally provide enough opportunity to complete the suggested ‘subjective impressions’ (Figure 1, purple) SLTs should aim to collect.

**TABLE 3:** Suggested stimulus tools and assessments for the core speech sample.

Speech area	Suggested tools for core speech sample
Initial screen	DEAP screen and the Intelligibility in Context Scale
Single word naming	DEAP phonology subtest or toddler version
Stimulability	Stimulability Assessment (Powell and Miccio 1996) or DEAP stimulability for consonants and vowels absent from the phonetic inventory
Intelligibility	Informal clinician rating based on connected speech from, for example, the Renfrew Action Picture Test, and the Intelligibility in Context Scale
(In)consistency	DEAP screen (repeated twice) then the DEAP inconsistency assessment if indicated

Abbreviation: DEAP = Diagnostic Evaluation of Articulation and Phonology.

### 3.10 Data Checking

A draft report, including the summary figures, was sent to participants for checking after the workshop. One participant reported that children might meet the criteria for phonological delay or consistent phonological disorder but be minimally stimulable for phonemes absent from their system (e.g., present with velar fronting yet are not stimulable for velar consonants) and therefore initially require an articulatory intervention approach. We suggest that these children do indeed receive a diagnosis of a phonological subtype of disorder, although some initial stimulability intervention may be required.

Three participants reported that there was ambiguity surrounding whether the DEAP inconsistency (Dodd et al. 2002) assessment was part of the core (Figure 1, blue) or drill down (Figure 1, red) assessment. A summary table (Table 3) clarifies that the DEAP screen includes an opportunity to sample the 10 words twice, and this should form part of the core assessment. Children who are more than 50% inconsistent should receive further inconsistency assessment as part of the ‘drill down’ assessment and in line with the DEAP manual instructions (Dodd et al. 2002).

One participant suggested that a specific vowel assessment might be a useful addition to the ‘drill down assessment’. This was added. A different participant suggested that core stimulability should also include /VCV/ stimulability. This was also added.

### 3.11 Stakeholder Engagement

Two hundred and eighty‐three participants registered for the free online CEN for SSD annual meeting. This meeting comprised several constituent regional networks which offer training and support for SLTs in the United Kingdom working with children with SSD. Registration for the event was capped at 300, and recordings were sent for distribution after the live meeting. Prior to the presentation of the above SSD definitions and protocol, participants were asked via an anonymous Zoom poll which classification system they individually used. Two hundred and four (204) participants responded to the poll: 55% use Dodd's Differential Diagnostic Classification System, 15% use the Psycholinguistic Framework, 0% use the SDCS, 2% use the ICF and 28% use a different system or no system. This corroborates the anecdotal evidence that most SLTs in the United Kingdom use Dodd's system and highlights the need for consensus given 28% use a different system or are unsure which system they are using.

Meeting participants then engaged with presentations on the above content analysis and participatory workshop results, including all figures presented here. Ninety‐three percent (93%) of participants agreed with the subtype definitions presented, and 87% of participants said they could implement them via the protocol ‘easily’. A further 13% of participants said they could implement them with difficulty, and fewer than 1% (only one participant) said they could not implement them.

## 4 Discussion

This study aimed to establish an initial consensus on classifying SSD subtypes and an agreed diagnostic protocol for arriving at these subtypes for SLTs working in the publicly funded NHS in the United Kingdom. We used participatory methods to arrive at agreed subtypes, with workable definitions, and a diagnostic protocol with suggested stimulus tools/assessments that would be feasible for clinicians and reflect current practice in the United Kingdom. Although an international consensus on SSD subtype labels and descriptions is desirable (Waring and Knight 2013), differences in the way SLT services are designed and funded, as well as the way SLTs are educated in different countries, makes this challenging, even within English‐speaking countries. Previous work has suggested that two main classification systems are in use internationally: the SDCS (Shriberg et al. 2010) and Dodd's Differential Diagnostic Classification System (Dodd 2014). Our content analysis of documents from services confirmed the anecdotal evidence that Dodd's system is preferred in the United Kingdom, and our anonymous poll corroborates this, although the psycholinguistic framework developed by Stackhouse and Wells (1993) was also used by a minority of participants. The choice of the Dodd (2014) system is likely due to the author having developed much of her work in the United Kingdom, therefore influencing university teaching on SSDs. Further, the availability of an assessment tool, the DEAP (Dodd et al. 2002) which maps directly to these subtypes and is standardised on an English‐speaking UK population, makes this system an obvious choice for the UK context. It is also worth noting that although the UK professional body, the RCSLT, does not specify a particular classification system that must be taught, the subtypes of SSD they suggest in their curriculum guidelines are broadly in line with the terms used by Dodd (2014).

### 4.1 SSD Terms and Definitions

Although the classification system was unanimously agreed upon, the group decided to change some of the subtype names and subsequently the definitions to reflect how these terms are operationalised in practice. While changing the diagnostic criteria for an SSD subtype might seem controversial, it is worth noting that diagnoses (especially those based on behavioural symptoms) do change over time, and our aim was to reflect how practitioners use diagnostic labels in real healthcare settings. First, CAS was chosen as the preferred term over Developmental Verbal Dyspraxia to reflect a growing international consensus that this term be used (Broomfield et al. 2022). Although the group opted to keep separate labels for phonological delay and consistent phonological disorder, there was considerable discussion over whether these should be subsumed into one label. Indeed, McLeod and Baker (2017) suggest one category of ‘phonological impairment’ to cover both phonological delay and disorder. This is because the intervention approaches for both are often the same, and the choice of intervention should be based more on the number of errors than the nature of these per se (Storkel 2022). To our knowledge there is no evidence in the current literature to support or specify a given maximum number of processes as a criterion for diagnosis of phonological delay, although the phrase ‘small number’ is used to suggest SSD treatment selection (Storkel 2022).

Maintaining this distinction could be important from a prognostic point of view and therefore prioritisation of services. In a longitudinal study of children between the ages of four and seven, Dodd et al. (2018) found that English‐speaking children with delayed phonological patterns were more likely to resolve (67%) than those with disordered patterns (35%) by age seven. However, in a large study of Cantonese‐speaking children, To et al. (2022) found that children with phonological delay did not necessarily resolve their speech quicker than those with phonological disorder. Given this, it seems wise to maintain a distinction if these labels are to be used in future research looking at response to intervention and prognosis in the United Kingdom. Moreover, the distinction between delay and disorder could be important for intervention choice. Recent work by Waring et al. (2022) suggests differences in cognitive flexibility between preschool children with delayed and disordered patterns. They suggest that intervention for children with phonological disorder should incorporate training in cognitive flexibility, though note this study compared only 13 children in each subtype and preschool children only. Given these points, it will be useful to maintain the distinction for evaluating interventions and for prioritisation of services.

In terms of changes to the definitions, some were minor clarifications, for example, clarifying that children with IPD should present with ‘*more than* or *equal to*’ 40% inconsistency and that “*sounds in isolation…during imitation*” refers to stimulability in the definition of articulation disorder. However, the group felt that it was important that the definition of phonological disorder be widened to include children with multiple phoneme collapses, that is, where one sound replaces many different sounds (Williams 2000), or older children with severe phonological delay. There was lengthy discussion about whether a ‘delay’ diagnosis is appropriate for children over 5 years. In part, a reluctance to use the ‘delay’ label may have been motivated by recent changes to the terminology for developmental language disorder. This definition change is at odds with Dodd's original work, where these children could be classified as having severe phonological delay, and we suggest that SLTs record whether children present with only delayed processes, even if they are many, or with additional disordered processes. A bigger change was made to the definition of CAS. Rather than remain consistent with the original definition given by Dodd (2014), they opted to adopt the definition given by the American Speech‐Language‐Hearing Association (2007). There has been an increase the number of treatment studies for CAS over the last decade, and most of these studies use the ASHA definition, making this a useful change.

### 4.2 Diagnostic Protocol

Researchers suggest that a thorough diagnostic protocol for SSD of unknown origin should include single‐word testing, additional single‐word testing, a connected speech sample, stimulability testing, and an assessment of inconsistency (Macrae 2016). Our participants suggested including all of these aspects in their core diagnostic protocol, with the exception of additional single‐word testing and the caveat that inconsistency should only be screened for all children and probed in depth for children who show evidence of a potential inconsistent phonological disorder. This contrasts with previous survey studies which suggest that some of these aspects are often missing in SLTs’ batteries of assessment (Diepeveen et al. 2020; McLeod and Baker 2014; Skahan et al. 2007). Our SLTs did agree that time and costs, especially the cost of assessments, are often a factor in choosing assessments (Diepeveen et al. 2020), and for this reason, there was a reluctance to suggest specific commercial assessments for collecting data. Indeed, Fabiano‐Smith (2019) suggests that commercial standardised assessments may be less accurate for differential diagnosis of SSD than criterion‐referenced measures, and it is certainly the case that a thorough analysis of a spontaneous speech sample can be diagnostically powerful (Bates et al. 2021). However, using an assessment such as the DEAP is likely to be much less time‐consuming, and therefore potentially cost‐saving in terms of staff time than analysing a connected speech sample. The DEAP (Dodd et al. 2002) was therefore suggested as a commercial assessment that could be recommended as a tool for gathering information about single‐word speech production, stimulability, and inconsistency. This is consistent with the choice of Dodd's Differential Diagnostic Classification System, as this test is specifically designed for differential diagnosis for the subtypes chosen by participants.

Although this study did not specifically set out to design a process only for children who speak English as their primary language, our UK focus arrived at a diagnostic protocol which utilises English‐language assessments. For children who do not speak English, the SLT will need to use equivalent tests developed for the target language, if available, or a spontaneous speech sample with assistance from an interpreter. There are still limitations even if these approaches are put in place. Many language‐specific assessments have been developed for children growing up in monolingual environments (McLeod and Verdon 2014) and interpreters would need specialist training to be able to identify specific errors in the speech of children with SSD. Evidence from cross‐linguistic studies is helpful, however, and tells us that most children have acquired the sound system of the languages they are exposed to by age five, with clear patterns regarding which consonants are acquired early and late in development according to manner and place characteristics (McLeod and Crowe 2018).

The participants also highlighted the importance of collecting a parent/carer measure of a child's perceived speech intelligibility. Participants suggested that the Intelligibility in Context Scale (McLeod et al. 2012) was both a useful method for screening for SSD (McLeod 2020) and for obtaining parents’/carers’ views on their child's intelligibility. The ICS is potentially also a useful measure of the severity of SSD. Patient (or in this case parent) reported outcome measures (PROMS) are relatively underused in speech and language therapy (Cohen and Hula 2020), and the inclusion of this measure highlights the importance of gathering the views of parents when assessing their child's speech. This assessment was also favoured because it is available in many languages, is free to use, and is quick to complete.

### 4.3 Limitations

This study is necessarily limited by its focus on the UK context. However, we argue that a similar process could be undertaken in other settings using as a starting point the subtype labels agreed here. Choice of assessments will be constrained by what is available in any particular country, in the correct language/s, and with relevant norms. A further limitation is our inclusion of only five services in the United Kingdom, represented by only six specialist SLTs. Although these services represent a variety of urban and rural locations, they covered only two (England and Scotland) of the four nations, and the more experienced SLTs may have been biased to designing a diagnostic protocol that was feasible for more experienced SLTs. This limitation is somewhat mitigated by our stakeholder engagement with the national (UK‐wide) CEN for SSDs, where our anonymous polls suggested overwhelming support for the subtype definitions and diagnostic protocol.

Although our choice of participatory design was well motivated by our desire to create an approach that could be implemented by clinicians immediately and with ease (Douglas et al. 2023), this has some drawbacks in terms of applying the latest evidence. Research suggests a research‐practice gap of up to 17 years (Morris et al. 2011), so it is not surprising that our content analysis suggested use of Dodd's Differential Diagnostic Classification System (2014), which was updated a decade ago and is based on work much older than this. However, recent work by Ttofari Eecen et al. (2019) does validate this model, with the addition of ‘suspected atypical speech motor control’ in the knowledge that children in this category (10% in their study) will require more in‐depth diagnostic procedures. Additionally, as new research findings become available on subtypes of SSD and diagnostic methods, this must be taken into account.

Lastly, we must acknowledge the focus here on SSD of unknown origin and of relatively straightforward presentation. While this does account for the majority of children with SSD, children with complex presentations may need additional assessment. For example, further assessment guided by a psycholinguistic framework (Geronikou and Rees 2016; Stackhouse and Wells 1993) was suggested by workshop participants as a supplementary method for pinpointing areas of difficulty for intervention planning. Moreover, children with SSDs of known origin, for example, SSD associated with cleft palate ± lip or Down syndrome, will also need a different approach, and children with suspected severe motor speech disorders, for example, concomitant CAS and childhood dysarthria, will need an in‐depth assessment of their speech strengths and weaknesses to allow treatment planning. Children with co‐occurring neurodiversities such as autism or developmental language disorder will also need careful consideration during the diagnostic process. While the current protocol suggests a variety of in‐depth diagnostic tests that could be applied to children with these more complex presentations, at present it is unclear how to determine if and when to undertake these assessments with specific children, the implication being that experienced clinicians will be able to determine this independently. It is worth, however, noting that the DEAP assessment (Dodd et al. 2002) does give guidance on when to use further assessment for children with suspected CAS. A future study seeks to trial the implementation of the diagnostic protocol with a large number of children with suspected SSD, and this iterative process will no doubt result in changes to the labels and/or diagnostic protocol and further guidance on if and when to undertake more in‐depth assessment.

## 5 Conclusions

This study sought to determine a common classification system and diagnostic protocol for childhood SSD of unknown origin using participatory methods. SLTs practising in the United Kingdom agreed that Dodd's classification system (Dodd 2014) was fit for purpose in the United Kingdom with some minor amendments to the descriptions of the subtypes and replacing ‘Developmental Verbal Dyspraxia’ with ‘Childhood Apraxia of Speech’ and adopting the ASHA definition of this SSD subtype. In terms of a diagnostic protocol, the participants agreed that a feasible protocol should include a minimum assessment required for children with more straightforward or mild SSD, with optional additional assessment for more complex cases. In conclusion, an initial consensus was reached on a classification system and diagnostic protocol for use in the United Kingdom.

## Ethics Statement

Ethical approval for this study was obtained from the study sponsor, Bristol NHS Trust. Full approval for the study was obtained from the NHS Health Research Authority (22/HRA/1962) prior to recruitment of participatory group members.

## Consent

There are no patient data in this study.

## Conflict of Interest

The authors declare no conflicts of interest.

## Data Availability

The data that support the findings of this study are available on request from the corresponding author. The data are not publicly available due to privacy or ethical restrictions.

## References

[jlcd70052-bib-0001] American Speech‐Language‐Hearing Association . 2007. Childhood Apraxia of Speech . Technical Report. ASHA.

[jlcd70052-bib-0002] Armstrong, S. , and M. Ainley . 1993. South Tyneside Assessment of Phonology. STASS. https://books.google.co.uk/books?id=QzKpAAAACAAJ.

[jlcd70052-bib-0003] Bates, S. , J. Titterington , and Child Speech Disorder Research Network . 2021. Good Practice Guidelines for the Analysis of Child Speech. Ulster University.

[jlcd70052-bib-0004] Bates, S. , and J. Watson . 2012. Phonetic and Phonological Systems Analysis (PPSA) . Queen Margaret University. https://www.qmu.ac.uk/schools‐and‐divisions/shs/ppsa/.

[jlcd70052-bib-0005] Bishop, D. V. M. , M. J. Snowling , P. A. Thompson , and T. Greenhalgh . 2016. “CATALISE: A Multinational and Multidisciplinary Delphi Consensus Study. Identifying Language Impairments in Children.” PLoS ONE 11, no. 7: e0158753. 10.1371/journal.pone.0158753. 27392128 PMC4938414

[jlcd70052-bib-0006] Broomfield, J. , J. Cleland , and P. Williams . 2022. “What's in a Name? Dr Jan Broomfield, Dr Joanne Cleland and Dr Pam Williams Make Their Case for Adopting the Term ‘Childhood Apraxia of Speech’.” Bulletin: The Official Magazine Of The Royal College Of Speech & Language Therapists no. 833: 1–2. https://strathprints.strath.ac.uk/83091/.

[jlcd70052-bib-0007] Broomfield, J. , and B. Dodd . 2004. “Children With Speech and Language Disability: Caseload Characteristics.” International Journal of Language & Communication Disorders 39, no. 3: 303–324. 10.1080/13682820310001625589 15204443

[jlcd70052-bib-0008] Broomfield, J. , and B. Dodd . 2005. “Clinical Effectiveness.” In Differential Diagnosis and Treatment of Children With Speech Disorder, edited by B. Dodd , 2nd ed., 211–229. Whurr.

[jlcd70052-bib-0010] Cohen, M. L. , and W. D. Hula . 2020. “Patient‐Reported Outcomes and Evidence‐Based Practice in Speech‐Language Pathology.” American Journal of Speech‐Language Pathology 29, no. 1: 357–370. 10.1044/2019_AJSLP-19-00076 32011905

[jlcd70052-bib-0011] Crosbie, S. L. , A. Holm , and B. Dodd . 2021. In Core Vocabulary Intervention, 2nd ed., edited by A. L. Williams , S. McLeod , and R. J. McCauley , 225–250. Paul H. Brookes Publishing Co. https://acuresearchbank.acu.edu.au/item/8xv7v/core‐vocabulary‐intervention. https://acuresearchbank.acu.edu.au/download/1d245965480c94e6d9c3a08b47396ea01197ba297d8c40b2705da5bb79bfcbcc/3146660/Crosbie_2021_Core_vocabulary_intervention.pdf.

[jlcd70052-bib-0012] Diepeveen, S. , H. Terband , L. van Haaften , et al. 2022. “Process‐Oriented Profiling of Speech Sound Disorders.” Children 9, no. 10: 1502. https://www.mdpi.com/2227‐9067/9/10/1502. 36291438 10.3390/children9101502 PMC9600371

[jlcd70052-bib-0013] Diepeveen, S. , L. Van Haaften , H. Terband , B. De Swart , and B. Maassen . 2020. “Clinical Reasoning for Speech Sound Disorders: Diagnosis and Intervention in Speech‐Language Pathologists' Daily Practice.” American Journal of Speech‐Language Pathology 29, no. 3: 1529–1549. 10.1044/2020_AJSLP-19-00040 32479738

[jlcd70052-bib-0014] Dodd, B. 2005. Differential Diagnosis and Treatment of Children With Speech Disorder. Wiley‐Blackwell.

[jlcd70052-bib-0015] Dodd, B. 2014. “Differential Diagnosis of Pediatric Speech Sound Disorder.” Current Developmental Disorders Reports 1, no. 3: 189–196. 10.1007/s40474-014-0017-3

[jlcd70052-bib-0016] Dodd, B. , K. Ttofari‐Eecen , K. Brommeyer , K. Ng , S. Reilly , and A. Morgan . 2018. “Delayed and Disordered Development of Articulation and Phonology Between Four and Seven Years.” Child Language Teaching and Therapy 34, no. 2: 87–99. 10.1177/0265659017735958.

[jlcd70052-bib-0017] Dodd, B. , H. Zhu , S. Crosbie , A. Holm , and A. Ozanne . 2002. Diagnostic Evaluation of Articulation and Phonology (DEAP). Psychological Corporation.

[jlcd70052-bib-0018] Douglas, N. , J. Hinckley , K. Grandbois , et al. 2023. “How a Power Differential Between Clinicians and Researchers Contributes to the Research‐to‐Practice Gap.” American Journal of Speech‐Language Pathology 32, no. 2: 803–810. 10.1044/2022_AJSLP-22-00207 36763851

[jlcd70052-bib-0019] Eadie, P. , A. Morgan , O. C. Ukoumunne , K. Ttofari Eecen , M. Wake , and S. Reilly . 2015. “Speech Sound Disorder at 4 Years: Prevalence, Comorbidities, and Predictors in a Community Cohort of Children.” Developmental Medicine & Child Neurology 57, no. 6: 578–584. 10.1111/dmcn.12635 25403868

[jlcd70052-bib-0020] Fabiano‐Smith, L. 2019. “Standardized Tests and the Diagnosis of Speech Sound Disorders.” Perspectives of the ASHA Special Interest Groups 4, no. 1: 58–66. 10.1044/2018_PERS-SIG1-2018-0018.

[jlcd70052-bib-0021] Geronikou, E. , and R. Rees . 2016. “Psycholinguistic Profiling Reveals Underlying Impairments for Greek Children With Speech Disorders.” Child Language Teaching and Therapy 32, no. 1: 95–110. 10.1177/0265659015583915.

[jlcd70052-bib-0022] Goldman, R. , and M. Fristoe . 2000. Goldman‐Fristoe Test of Articulation, 2nd ed. American Guidance Service.

[jlcd70052-bib-0023] Harding, S. , S. Burr , J. Cleland , H. Stringer , and Y. Wren . Unpublished manuscript. “Children With Speech Sound Disorder: Emotional Mapping to Understand Their Experiences of Speech and Language Therapy Intervention.” Child Language Teaching and Therapy .

[jlcd70052-bib-0024] Hsieh, H.‐F. , and S. E. Shannon . 2005. “Three Approaches to Qualitative Content Analysis.” Qualitative Health Research 15, no. 9: 1277–1288. 10.1177/1049732305276687. 16204405

[jlcd70052-bib-0025] Kerryjane, M. , and K. Spilsby . 2006. CLEAR: Phonology Screening Assessment. Clear Resources.

[jlcd70052-bib-0026] Macrae, T. 2016. “Comprehensive Assessment of Speech Sound Production in Preschool Children.” Perspectives of the ASHA Special Interest Groups 1, no. 1: 39–56. 10.1044/persp1.SIG1.39.

[jlcd70052-bib-0027] Maitland, M. E. 2010. “A Transdisciplinary Definition of Diagnosis.” Journal of Allied Health 39, no. 4: 306–313. 21184028

[jlcd70052-bib-0028] McIntosh, B. , and B. Dodd . 2011. Toddler Phonology Test. Pearson.

[jlcd70052-bib-0029] McLeod, S. 2020. “Intelligibility in Context Scale: Cross‐linguistic Use, Validity, and Reliability.” Speech, Language and Hearing 23, no. 1: 9–16. 10.1080/2050571X.2020.1718837. 22215036

[jlcd70052-bib-0030] McLeod, S. , and E. Baker . 2014. “Speech‐Language Pathologists' Practices Regarding Assessment, Analysis, Target Selection, Intervention, and Service Delivery for Children With Speech Sound Disorders.” Clinical Linguistics & Phonetics 28, no. 7–8: 508–531. 10.3109/02699206.2014.926994. 25000375

[jlcd70052-bib-0031] McLeod, S. , and E. Baker . 2017. Children's Speech: an Evidence‐based Approach to Assessment and Intervention. Pearson.

[jlcd70052-bib-0032] McLeod, S. , and K. Crowe . 2018. “Children's Consonant Acquisition in 27 Languages: A Cross‐Linguistic Review.” American Journal of Speech‐Language Pathology 27, no. 4: 1546–1571. 10.1044/2018_AJSLP-17-0100. 30177993

[jlcd70052-bib-0033] McLeod, S. , L. J. Harrison , and J. McCormack . 2012. “The Intelligibility in Context Scale: Validity and Reliability of a Subjective Rating Measure.” Journal of Speech, Language, and Hearing Research 55, no. 2: 648–656. 10.1044/1092-4388(2011/10-0130). 22215036

[jlcd70052-bib-0034] McLeod, S. , and S. Verdon . 2014. “A Review of 30 Speech Assessments in 19 Languages Other than English.” American Journal of Speech‐Language Pathology 23, no. 4: 708–723. 10.1044/2014_AJSLP-13-0066. 24700105

[jlcd70052-bib-0035] Miccio, A. W. , and A. L. Williams . 2021. “Stimulability Approach.” In Interventions for Speech Sound Disorders in Children, 2nd ed., edited by A. L. Williams , S. McLeod , and R. J. McCauley , 279–304. Paul H. Brookes Publishing Co.

[jlcd70052-bib-0036] Michie, S. , L. Atkins , and R. West . 2014. The Behaviour Change Wheel. A Guide to Designing Interventions. Silverback Publishing.

[jlcd70052-bib-0037] Morris, Z. S. , S. Wooding , and J. Grant . 2011. “The Answer Is 17 Years, What Is the Question: Understanding Time Lags in Translational Research.” Journal of the Royal Society of Medicine 104, no. 12: 510–520. 10.1258/jrsm.2011.110180. 22179294 PMC3241518

[jlcd70052-bib-0038] Powell, T. W. , and A. W. Miccio . 1996. “Stimulability: A Useful Clinical Tool.” Journal of Communication Disorders 29, no. 4: 237–253. 10.1016/0021-9924(96)00012-3. 8863117

[jlcd70052-bib-0039] RCSLT . 2021. “Curriculum Guidance.” Last modified March 2021. https://www.rcslt.org/wp‐content/uploads/2020/08/RCSLT‐Curriculum‐Guidance‐March2021.pdf.

[jlcd70052-bib-0040] Renfrew, C. E. 2016. The Renfrew Language Scales: Action Picture Test. Speechmark.

[jlcd70052-bib-0041] Roper, A. , and J. Skeat . 2022. “Innovation Through Participatory Design: Collaborative Qualitative Methods in the Development of Speech‐Language Pathology Technology.” International Journal of Speech‐Language Pathology 24, no. 5: 527–532. 10.1080/17549507.2022.2050943. 35506478

[jlcd70052-bib-0042] Sell, D. , A. Harding , and P. Grunwell . 1999. “GOS.SP.ASS.'98: An Assessment for Speech Disorders Associated With Cleft Palate and/or Velopharyngeal Dysfunction (Revised).” International Journal of Language & Communication Disorders 34, no. 1: 17–33. 10.1080/136828299247595. 10505144

[jlcd70052-bib-0043] Shriberg, L. D. , M. Fourakis , S. D. Hall , et al. 2010. “Extensions to the Speech Disorders Classification System (SDCS).” Clinical Linguistics & Phonetics 24, no. 10: 795–824. 10.3109/02699206.2010.503006. 20831378 PMC2941221

[jlcd70052-bib-0044] Shriberg, L. D. , E. A. Strand , K. J. Jakielski , and H. L. Mabie . 2019. “Estimates of the Prevalence of Speech and Motor Speech Disorders in Persons With Complex Neurodevelopmental Disorders.” Clinical Linguistics & Phonetics 33, no. 8: 707–736. 10.1080/02699206.2019.1595732. 31221012 PMC6633911

[jlcd70052-bib-0045] Skahan, S. M. , M. Watson , and G. L. Lof . 2007. “Speech‐Language Pathologists' Assessment Practices for Children with Suspected Speech Sound Disorders: Results of a National Survey.” American Journal of Speech‐Language Pathology 16, no. 3: 246–259. 10.1044/1058-0360(2007/029). 17666550

[jlcd70052-bib-0046] Stackhouse, J. , and B. Wells . 1993. “Psycholinguistic Assessment of Developmental Speech Disorders.” European Journal of Disorders of Communication 28, no. 4: 331–348. 10.3109/13682829309041469. 8312650

[jlcd70052-bib-0047] Storkel, H. L. 2022. “Minimal, Maximal, or Multiple: which Contrastive Intervention Approach to Use with Children with Speech Sound Disorders?” Language, Speech, and Hearing Services in Schools 53, no. 3: 632–645. 10.1044/2021_LSHSS-21-00105. 35179980

[jlcd70052-bib-0048] Terband, H. , B. Maassen , and E. Maas . 2019. “A Psycholinguistic Framework for Diagnosis and Treatment Planning of Developmental Speech Disorders.” Folia Phoniatrica Et Logopaedica 71, no. 5–6: 216–227. 10.1159/000499426. 31269495 PMC7050667

[jlcd70052-bib-0049] To, C. K. S. , S. McLeod , K. L. Sam , and T. Law . 2022. “Predicting which Children Will Normalize Without Intervention for Speech Sound Disorders.” Journal of Speech, Language, and Hearing Research 65, no. 5: 1724–1741. 10.1044/2022_JSLHR-21-00444. 35381182

[jlcd70052-bib-0050] Ttofari Eecen, K. , P. Eadie , A. T. Morgan , and S. Reilly . 2019. “Validation of Dodd's Model for Differential Diagnosis of Childhood Speech Sound Disorders: A Longitudinal Community Cohort Study.” Developmental Medicine & Child Neurology 61, no. 6: 689–696. 10.1111/dmcn.13993. 30151900

[jlcd70052-bib-0051] Waring, R. , and R. Knight . 2013. “How Should Children With Speech Sound Disorders be Classified? A Review and Critical Evaluation of Current Classification Systems.” International Journal of Language & Communication Disorders 48, no. 1: 25–40. 10.1111/j.1460-6984.2012.00195.x. 23317382

[jlcd70052-bib-0052] Waring, R. , S. Rickard Liow , B. Dodd , and P. Eadie . 2022. “Differentiating Phonological Delay From Phonological Disorder: Executive Function Performance in Preschoolers.” International Journal of Language & Communication Disorders 57, no. 2: 288–302. 10.1111/1460-6984.12694 35060663

[jlcd70052-bib-0053] Williams, A. L. 2000. “Multiple Oppositions.” American Journal of Speech‐Language Pathology 9, no. 4: 282–288. 10.1044/1058-0360.0904.282.

[jlcd70052-bib-0054] Williams, P. , and H. Stephens . 2004. Nuffield Centre Dyspraxia Programme. Miracle Factory.

[jlcd70052-bib-0055] Wren, Y. , S. Harding , J. Goldbart , and S. Roulstone . 2018. “A Systematic Review and Classification of Interventions for Speech‐Sound Disorder in Preschool Children.” International Journal of Language & Communication Disorders 53, no. 3: 446–467. 10.1111/1460-6984.12371. 29341346

[jlcd70052-bib-0056] Wren, Y. , L. L. Miller , T. J. Peters , A. Emond , and S. Roulstone . 2016. “Prevalence and Predictors of Persistent Speech Sound Disorder at Eight Years Old: Findings from a Population Cohort Study.” Journal of Speech, Language, and Hearing Research 59, no. 4: 647–673. 10.1044/2015_JSLHR-S-14-0282. PMC5280061 27367606

[jlcd70052-bib-0057] Wren, Y. , E. Pagnamenta , F. Orchard , et al. 2023. “Social, Emotional and Behavioural Difficulties Associated With Persistent Speech Disorder in Children: A Prospective Population Study.” JCPP Advances 3, no. 1: e12126. 10.1002/jcv2.12126. 37431315 PMC10241475

[jlcd70052-bib-0058] Wren, Y. , E. Pagnamenta , T. J. Peters , et al. 2021. “Educational Outcomes Associated With Persistent Speech Disorder.” International Journal of Language & Communication Disorders 56, no. 2: 299–312. 10.1111/1460-6984.12599. 33533175 PMC8591628

